# Software-based quantitative CT analysis to predict the growth trend of persistent nonsolid pulmonary nodules: a retrospective study

**DOI:** 10.1007/s11547-023-01648-z

**Published:** 2023-05-25

**Authors:** Andrea Borghesi, Felice Leopoldo Coviello, Alessandra Scrimieri, Pietro Ciolli, Marco Ravanelli, Davide Farina

**Affiliations:** Department of Medical and Surgical Specialties, Radiological Sciences and Public Health, University of Brescia, ASST Spedali Civili of Brescia, Piazzale Spedali Civili, 1, 25123 Brescia, Italy

**Keywords:** Pulmonary nodule, Subsolid nodule, Nonsolid nodule, Pure ground-glass nodule, Computed tomography, Computer-assisted image analysis

## Abstract

**Purpose:**

Persistent nonsolid nodules (NSNs) usually exhibit an indolent course and may remain stable for several years; however, some NSNs grow quickly and require surgical excision. Therefore, identifying quantitative features capable of early discrimination between growing and nongrowing NSNs is becoming a crucial aspect of radiological analysis. The main purpose of this study was to evaluate the performance of an open-source software (ImageJ) to predict the future growth of NSNs detected in a Caucasian (Italian) population.

**Material and methods:**

We retrospectively selected 60 NSNs with an axial diameter of 6–30 mm scanned with the same acquisition-reconstruction parameters and the same computed tomography (CT) scanner. Software-based analysis was performed on thin-section CT images using ImageJ. For each NSNs, several quantitative features were extracted from the baseline CT images. The relationships of NSN growth with quantitative CT features and other categorical variables were analyzed using univariate and multivariable logistic regression analyses.

**Results:**

In multivariable analysis, only the skewness and linear mass density (LMD) were significantly associated with NSN growth, and the skewness was the strongest predictor of growth. In receiver operating characteristic curve analyses, the optimal cutoff values of skewness and LMD were 0.90 and 19.16 mg/mm, respectively. The two predictive models that included the skewness, with or without LMD, exhibited an excellent power for predicting NSN growth.

**Conclusion:**

According to our results, NSNs with a skewness value > 0.90, specifically those with a LMD > 19.16 mg/mm, should require closer follow-up due to their higher growth potential, and higher risk of becoming an active cancer.

## Introduction

Pulmonary subsolid nodules (SSNs) are a relatively frequent finding on chest computed tomography (CT) examinations performed both in routine clinical practice and in lung cancer screening programs [1–4]. SSNs correspond to nodular pulmonary opacities of heterogeneous density that do not completely hide the underlying lung structure [5]. On CT images, SSNs are classified into two different subgroups depending on the presence or absence of an intralesional solid component [5–7]. SSNs with a solid component are conventionally called part-solid nodules (PSNs), while those without a solid component are called nonsolid nodules (NSNs) or pure ground-glass nodules, based on the authors’ preferences [1–7].

SSNs can be transient (i.e., nodules that disappear on follow-up CT scans) or persistent (i.e., nodules that persist on follow-up CT scans after an interval of ≥ 3 months) [5–11]. While transient SSNs represent benign lesions (most often inflammatory lesions) [9, 10], persistent SSNs (both NSNs and PSNs) are frequently the manifestation of preinvasive or invasive lung lesions, usually lung adenocarcinomas [12–14], and rarely other pulmonary malignancies, such as lymphomas or metastases [15–18].

Persistent SSNs exhibit different patterns of growth: most nodules (primarily NSNs) remain stable for many years or grow in an extremely slow way, with a doubling time (DT) that can exceed 6 years, whereas others grow faster, particularly PSNs with a predominantly solid intralesional component, with a DT of less than 1 year [12, 13].

Based on their indolent course and long DT, persistent NSNs are considered lesions with a very low likelihood of becoming a clinically active cancer. Therefore, regardless of the clinical setting of presentation, current guidelines recommend conservative management for NSNs [6, 7, 19]. Specifically, the Fleischner Society guidelines for nodules detected in clinical routine in patients aged ≥ 35 years suggest a first follow-up chest CT scan at 6 – 12 months only for NSNs ≥ 6 mm in size and subsequent biennial follow-up until 5 years [6, 7], whereas the Lung CT Screening Reporting and Data System guidelines for nodules detected in lung cancer screening programs recommend annual low-dose chest CT screening for any NSNs < 30 mm in size [19].

However, not all NSNs have a benign behavior and can be managed conservatively [5, 20]. In fact, persistent NSNs could exhibit a more aggressive behavior on follow-up CT scans by developing an intralesional solid component and progressively increasing the growth rate, which would inevitably require surgical resection [13]. Thus, predicting the future growth of persistent NSNs from CT images remains a challenging diagnostic goal. Although prior studies have reported that software-based quantitative CT analysis can be effective in predicting the future growth of persistent NSNs [5, 21–27], many of these software-based methods are not easily accessible to everyone because they are commercial, home-built, or require skills that not everyone possesses [22, 23, 25–27].

To our knowledge, only one study conducted on Asian (Chinese) patients has tested the performance of an open-source software (3D Slicer) for predicting the growth of persistent NSNs [21]. Considering the differences in the biology of persistent SSNs between Asian and Caucasian populations [28], the main purpose of our study was to investigate the performance of another open-source software (ImageJ) to predict the future growth of NSNs with an axial diameter of 6 − 30 mm detected in a Caucasian (Italian) population.

## Materials and methods

This retrospective study was notified to our local ethics committee and authorized by our university and hospital authorities (Protocol No. 15918, February 26, 2019). All procedures performed in this study were in conformity with the ethical standards and the 2013 version of the Declaration of Helsinki.

### Patient and nodule selection

We retrospectively reviewed data from our radiology information system and picture archiving and communication system for the period between January 2011 and February 2019. Patients and nodules were enrolled using a multi-step method. First, we retrieved all chest CT reports including radiological descriptive terms suggestive of NSNs (NSN, SSN, pure ground-glass nodule, and ground-glass nodule). Next, we reviewed the related images to check for persistent NSNs. Finally, patients and persistent NSNs were selected based on the following criteria: (1) Caucasian (Italian) ethnicity, (2) patients aged ≥ 35 years, (c) patients without history of steroid or chemotherapy treatment, (4) patients without a recent history of inflammatory or infectious lung disease, (5) no evidence of interstitial lung disease on CT images, (6) solitary NSNs with a mean diameter of 6 − 30 mm on axial CT images, (7) two or more chest CT scans performed with the same acquisition parameters and the same CT scanner, (8) unenhanced chest CT images reconstructed as a 1-mm-thick sections with lung window settings using the same sharp reconstruction algorithm, and (9) no evidence of motion or respiratory artifacts on chest CT images.

### CT image acquisition

Chest CT scans were performed using a 128 detector-raw CT scanner (Somatom Definition Flash, Siemens Healthineers, *Forchheim,* Germany) with predefined acquisition parameters as follows: tube voltage, 120 kVp; tube current–time product, 110 mAs with activated automatic exposure control system; and beam pitch, 1.2. Chest CT images were obtained at full inspiration without a spirometric gating system. Lung window CT images were reconstructed in the transverse plane as 1-mm-thick images using a sharp reconstruction algorithm.

### CT image analysis

Axial thin-section CT images with lung window settings were initially analyzed to assess the NSN location and check for the presence of emphysema in the lung parenchyma. Subsequently, both baseline and last follow-up CT images containing NSNs were sent to and processed using an open-source software (ImageJ 1.53c, Wayne Rasband, National Institutes of Health, USA) [29].

**Fig. 1:**
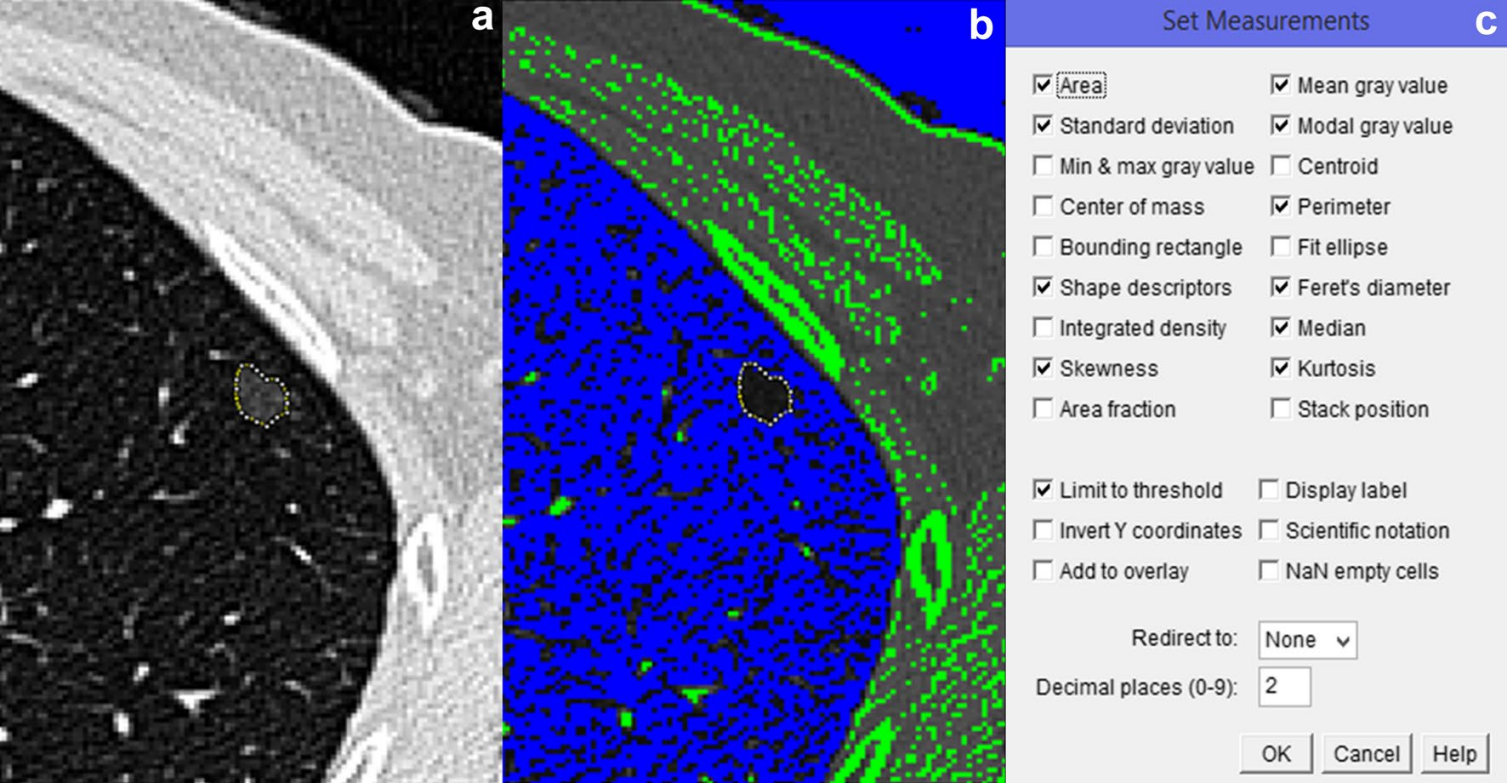
Images showing a summary of the steps of software-based analysis using ImageJ in a nonsolid nodule (NSN): **a** polygonal region of interest outlining the NSN contours, **b** threshold image obtained by applying an attenuation value of − 800 HU suitable for NSN segmentation, **c** list of quantitative CT features selected for NSN analysis

Segmentation and quantitative analysis of the selected NSNs were performed on the largest cross-sectional areas of the nodules, as described in a previous study on PSNs [30] (Fig. 1).

The following 12 quantitative CT features were selected for NSN analysis on baseline CT images: (1) Feret’s diameter (i.e., the mean of the maximum and minimum Feret’s diameters), (2) perimeter, (3) area, (4) mean CT attenuation, (5) median CT attenuation, (6) modal CT attenuation, (7) standard deviation of CT attenuation, (8) linear mass density (LMD), (9) skewness of CT attenuation, (10) kurtosis of CT attenuation, (11) circularity and (12) solidity.

As previously reported [30], LMD corresponds to the mass per unit length and was calculated as follows: [area × (mean CT attenuation + 1,000)]/1,000]. Circularity and solidity are dimensionless shape descriptors that provide information regarding the shape of a nodule and can take values ranging from 1 to 0. A circularity value of 1 indicates that an NSN has a shape similar to a perfect circle, and a solidity value of 1 indicates that the nodule edges are smooth and sharp, without indentations.

To determine the reliability of the selected quantitative CT features, the software-based analysis of the NSNs on baseline CT images was repeated after an interval of at least three months. For each quantitative CT features, the average value between the two measurements was used in this study.

To quantify the growth of NSNs, we calculated the DT of each nodule by matching the LMD obtained on baseline and last follow-up chest CT examinations. NSNs with a DT less than the cutoff value of 1,556 days were defined as growing nodules [12]. In the last follow-up CT scans, the development of a solid component within the NSNs was also evaluated.

Image analysis was performed independently by a thoracic radiologist (A.B.) with 16 years of experience in chest CT imaging and 11 years of experience in using ImageJ software.

### Statistical analysis

In this study, data distribution was verified by means of the Shapiro–Wilk test. While normally distributed data are presented as mean and standard deviation values, nonnormally distributed data are presented as median values and interquartile ranges (IQRs).

To determine the reliability of the selected quantitative CT features, the two sets of measurements obtained from the baseline CT images were compared using the intraclass correlation coefficient (ICC).

Spearman’s rank correlation analysis was used to assess the correlations between NSN DT and the selected quantitative CT features measured on baseline CT images. We used this nonparametric test because data related to the quantitative features and NSN DT were not normally distributed. The Mann–Whitney *U*-test was applied to analyze the differences in the quantitative features between growing (DT < 1,556 days) and nongrowing NSNs (DT ≥ 1,556 days).

The relationship of NSN growth with quantitative CT features and other categorical variables (i.e., patient age, sex, smoking habits, history of cancer, pulmonary emphysema, and NSN location) was analyzed using univariate and multivariable logistic regression analyses using a backward stepwise method. The power of the independent predictive factors for NSN growth was expressed as the area under the curve (AUC). Statistical analysis was performed using a commercial statistical software (MedCalc Software Ltd., version 20.114, Ostend, Belgium; https://www.medcalc.org; 2022). Statistical significance was set at *p* < 0.05.

## Results

According to the study selection criteria, 60 Caucasian (Italian) patients aged ≥ 35 years with a solitary NSN were enrolled in this retrospective analysis. Chest CT scans were performed for cancer follow-up in 29/60 (48.3%) patients and in the remaining 31/60 (51.7%) patients for other clinical indications (such as pulmonary opacity on chest radiograph, chronic obstructive pulmonary disease, suspected bronchiectasis, persistent cough or shortness of breath). The characteristics of the study patients are listed in Table 1.

**Table 1:** Characteristics of the study patients (*n* = 60)

Characteristics	
Age (years)	64.6 ± 11.4
Sex	
Male	25 (41.7)
Female	35 (58.3)
Smoking habits	
Current or former	24 (40.0)
Never	36 (60.0)
History of cancer	29 (48.3)
Lung cancer	10 (34.5)
Breast cancer	5 (17.2)
Malignant melanoma	4 (13.8)
Soft tissue sarcoma	4 (13.8)
Other malignancy	6 (20.7)

Data are presented as the number (percentage) or mean ± standard deviation

In the preliminary analysis of the thin-section CT images, pulmonary emphysematous changes were detected in 9 (15.0%) patients. The locations of the 60 NSNs were as follows: 23 (38.3%) in the right upper lobe, 22 (36.7%) in the left upper lobe, 6 (10.0%) in the right middle lobe, 3 (5.0%) in the right lower lobe, and 6 (10.0%) in the left lower lobe.

Software-based quantification analysis was successfully performed for all 60 NSNs on both baseline and last follow-up chest CT examinations. On baseline CT images, the ICC values (median, 0.88; IQR, 0.84–0.97) indicated an excellent or good reliability of the measurements for all quantitative CT features.

The median time interval between the first and last CT examinations was 1,136 days (IQR, 748 − 1,818 days). The median DT of NSNs with a positive variation in LMD between the first and last CT images was 2,816 days (IQR, 986 − 7,472 days).

Spearman’s rank correlation analysis showed that the following quantitative CT features were significantly correlated with the NSN DT: Feret’s diameter, perimeter, area, LMD, skewness of CT attenuation, kurtosis of CT attenuation, circularity, and solidity (*p* ≤ 0.002 for all). The results are summarized in Table 2.

**Table 2:** Correlation between quantitative features and doubling time of the 60 nonsolid nodules

Quantitative features	Value	Spearman’s Rho*	*p* Value*
Feret’s diameter (mm)†	8.27 (6.79–11.45)	− 0.535	< 0.001
Perimeter (mm)	27.04 (21.89–37.21)	− 0.572	< 0.001
Area (mm^2^)	38.74 (26.44–69.14)	− 0.519	< 0.001
Mean CT attenuation (HU)	− 611.59 (− 650.72– − 560.24)	− 0.028	0.830
Median CT attenuation (HU)	− 637.25 (− 673.50– − 594.00)	0.023	0.863
Modal CT attenuation (HU)	− 746.00 (− 765.50– − 658.00)	0.119	0.365
SD of CT attenuation (HU)	130.17 (107.54–163.29)	− 0.207	0.113
LMD (mg/mm)	14.69 (10.07–25.31)	− 0.499	< 0.001
Skewness of CT attenuation	0.80 (0.57–1.11)	− 0.498	< 0.001
Kurtosis of CT attenuation	0.31 (− 0.33–0.99)	− 0.396	0.002
Circularity	0.69 (0.57–0.76)	0.509	< 0.001
Solidity	0.82 (0.70–0.87)	0.407	0.001

Data are presented as the median (interquartile range)

*SD* Standard deviation, *LMD* Linear mass density

^†^Average of maximum and minimum Feret’s diameters

^*^Data obtained from the Spearman’s rank correlation analysis

Significant growth (DT < 1,556 days) was observed in 19 (31.7%) NSNs, of which 10 (52.6%) developed a small intralesional solid component (less than 6 mm) and 7 (36.8%) were surgically excised with a histopathological diagnosis of lung adenocarcinoma (three minimally invasive and four invasive adenocarcinomas). The quantitative features measured on baseline CT images of both growing and nongrowing NSNs are listed in Table 3.

**Table 3:** Quantitative features of growing and nongrowing NSNs on baseline CT images

Quantitative features	Growing NSNs (*n* = 19)	Nongrowing NSNs (*n* = 41)	*p* value*
Feret’s diameter (mm)†	11.45 (9.91–14.78)	7.20 (6.43–9.13)	< 0.001
Perimeter (mm)	38.32 (33.62–50.03)	23.01 (20.76–28.28)	< 0.001
Area (mm^2^)	70.64 (47.89–88.40)	32.17 (23.71–49.80)	< 0.001
Mean CT attenuation (HU)	− 609.27 (− 639.29– − 556.83)	− 631.60 (− 667.59– − 562.70)	0.530
Median CT attenuation (HU)	− 633.00 (− 661.50– − 600.75)	− 639.50 (− 690.88– − 577.13)	0.962
Modal CT attenuation (HU)	− 752.25 (− 765.50– − 692.25)	− 738.00 (− 768.50– − 641.00)	0.520
SD of CT attenuation (HU)	148.04 (129.72–175.81)	121.01 (102.86–156.33)	0.054
LMD (mg/mm)	26.63 (19.60–40.23)	12.81 (9.21–17.13)	< 0.001
Skewness of CT attenuation	1.12 (0.93–1.32)	0.65 (0.46–0.84)	< 0.001
Kurtosis of CT attenuation	1.19 (0.54–1.84)	− 0.08 (− 0.41–0.61)	< 0.001
Circularity	0.56 (0.55–0.67)	0.72 (0.65–0.78)	< 0.001
Solidity	0.71 (0.65–0.80)	0.83 (0.75–0.88)	0.003

Data are presented as the median (interquartile range)

*NSNs* Nonsolid nodules, *SD* Standard deviation, *LMD* Linear mass density

^†^Average of maximum and minimum Feret’s diameters

^*^p Values obtained by means the Mann–Whitney *U*-test

A statistically significant difference between growing and nongrowing NSNs was observed for the Feret’s diameter, perimeter, area, LMD, skewness of CT attenuation, kurtosis of CT attenuation, circularity, and solidity (*p* ≤ 0.003 for all).

The results of the univariate and multivariable logistic regression analyses for the relationship of NSN growth with quantitative CT features and the other categorical variables (patient age, sex, smoking habits, history of cancer, pulmonary emphysema, and NSN location) are presented in Table 4.

**Table 4:** Univariate and multivariable logistic regression analysis for predictors of nodule growth

Quantitative and categorical features	Univariate analysis			Multivariable analysis		
	OR	*β*	*p* value	OR	*β*	*p* value
Feret’s diameter (mm)†	1.634	0.491	< 0.001			
Perimeter (mm)	1.165	0.153	< 0.001			
Area (mm^2^)	1.052	0.050	< 0.001			
Mean CT attenuation (HU)	1.002	0.002	0.599			
Median CT attenuation (HU)	1.000	− 0.001	0.940			
Modal CT attenuation (HU)	0.997	− 0.003	0.503			
SD of CT attenuation (HU)	1.011	0.011	0.120			
LMD (mg/mm)	1.116	0.109	< 0.001	1.145	0.136	0.002
Skewness of CT attenuation	39.479	3.676	< 0.001	122.528	4.808	0.002
Kurtosis of CT attenuation	2.051	0.718	0.009			
Circularity	< 0.001	− 9.352	0.001			
Solidity	< 0.001	− 8.360	0.004			
Patient age (years)	1.064	0.062	0.040			
Sex^§^ (male vs female)	1.406	0.341	0.543			
Smoking habits^§^ (current/former vs never)	2.962	1.086	0.058			
History of cancer^§^ (yes vs no)	0.945	− 0.057	0.919			
Emphysema^§^ (yes vs no)	11.375	2.431	0.005			
Nodule location^§^ (upper vs middle/inferior lobes)	1.375	0.318	0.632			

*OR* Odds ratio, *β* Regression coefficient, *SD* Standard deviation, *LMD* Linear mass density

^†^Average of maximum and minimum Feret’s diameters

^§^Categorical variables

**Fig. 2:**
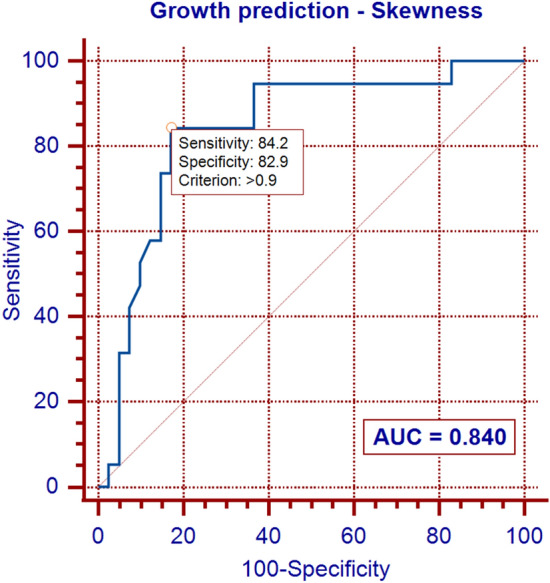
ROC curve for nonsolid nodule growth: model based on skewness of CT attenuation

**Fig. 3:**
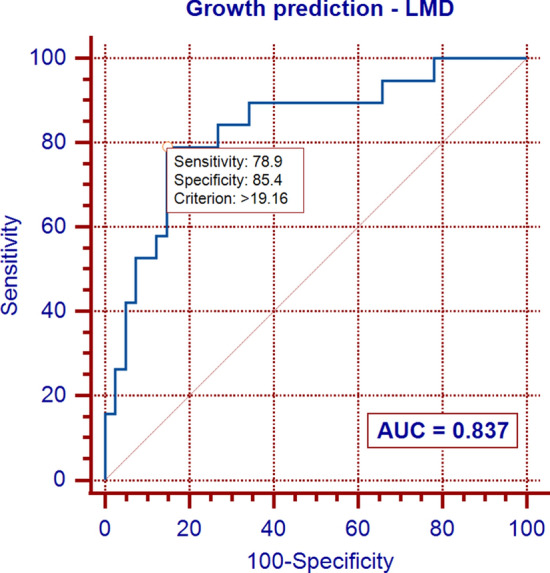
ROC curve for nonsolid nodule growth: model based on linear mass density (LMD)

**Fig. 4:**
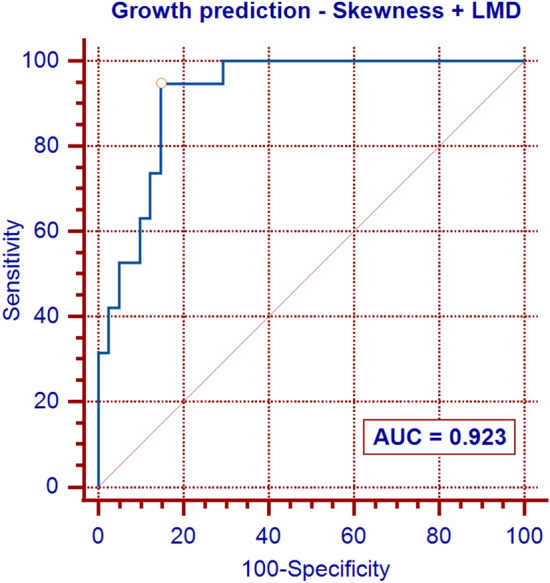
ROC curve for nonsolid nodule growth: model based on skewness of CT attenuation and linear mass density (LMD)

In the univariate analysis, several quantitative features (Feret’s diameter, perimeter, area, LMD, skewness of CT attenuation, kurtosis of CT attenuation, circularity, and solidity), patient age, and pulmonary emphysema were significantly associated with NSN growth (*p* ≤ 0.040 for all). However, in the multivariable analysis using a backward stepwise method, only the skewness of CT attenuation and the LMD were found to be independent predictors of NSN growth (Table 4). In receiver operating characteristic curve analyses, the optimal cutoff values of skewness and LMD were 0.90 and 19.16 mg/mm, respectively (Figs. 2, and 3). The two predictive models that included the skewness, with or without LMD, exhibited an excellent power for predicting NSN growth with AUC values of 0.923 and 0.840, respectively (Figs. 2, and 4).

## Discussion

The progressive improvement and increased availability of software-based methods for quantitative analysis of radiological images are driving a significant shift in the way radiologists analyze images. The application of such methods on chest CT images is constantly expanding, particularly in the management and risk stratification of lung nodules (both solid and subsolid) [4, 5, 12, 13, 21–27, 30–41].

Currently, software-based analysis of lung nodules is very promising and is attracting great interest, not only among thoracic radiologists but also among other thoracic physicians. Among software-based methods applied to SSNs, those related to the prediction of histological invasiveness of resected SSNs are widely described in the literature [35–39], whereas those related to the prediction of growth are few and have been investigated almost exclusively in Asian populations [22–27, 30, 41].

To our knowledge, the present study is the first to evaluate the performance of a software-based method for predicting the growth trend in solitary NSNs with a diameter of 6 − 30 mm in a Caucasian (Italian) population.

**Fig. 5:**
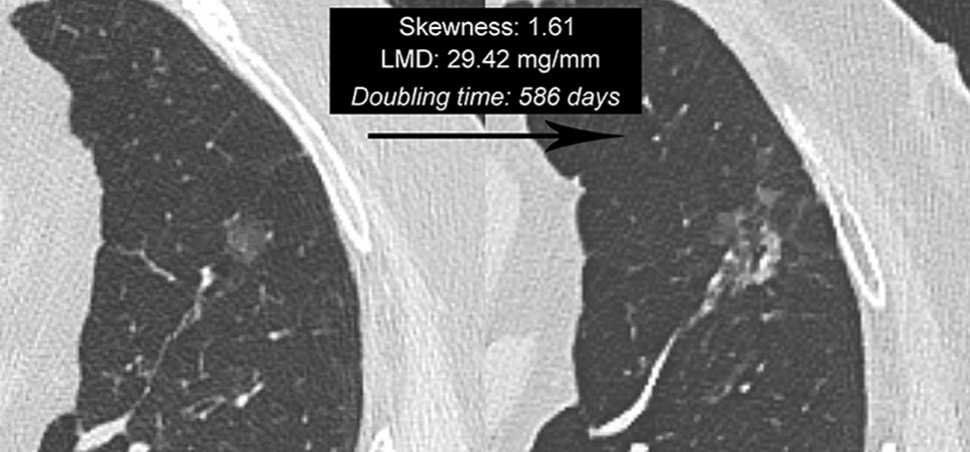
Growing nonsolid nodule (doubling time of 586 days) in the left upper lobe in a 71-year-old man, former smoker with no previous oncologic history. The interval between the baseline (*left*) and the last follow-up CT examination (*right*) was 1020 days. The software-based analysis performed on the baseline CT images obtained a skewness value of 1.61 and a LMD of 29.42 mg/mm. This nodule was surgically excised with a histopathological diagnosis of invasive pulmonary adenocarcinoma graded as pT1cN0

In our study, we tested the predictive value of several quantitative CT features such as Feret’s diameter, perimeter, area, mean CT attenuation, median CT attenuation, modal CT attenuation, standard deviation of CT attenuation, LMD, skewness of CT attenuation, kurtosis of CT attenuation, shape descriptors (circularity and solidity). These quantitative CT features were extracted from the largest cross-sectional areas of the NSNs on baseline CT images using an open-source software (ImageJ). From the data obtained using the ImageJ software, we found a significant correlation between NSN growth and the following CT features: Feret’s diameter, perimeter, area, LMD, skewness of CT attenuation, kurtosis of CT attenuation, circularity, and solidity. This significant correlation was found both by considering the absolute DT value and by comparing the groups of growing and nongrowing NSNs based on the DT cutoff value of 1,556 days. The univariate analysis confirmed the value of the same CT features for predicting future NSN growth; however, in the multivariate analysis, only the skewness of CT attenuation and the LMD were independent predictors for NSN growth. Specifically, a positive skewness value greater than 0.90 and a LMD greater than 19.16 mg/mm were significantly associated with NSN growth (Fig. 5).

In our study, the skewness of CT attenuation was found to be the strongest predictor of growth, and the two models that included the skewness, with or without LMD, showed an excellent predictive power for NSN growth.

Skewness of CT attenuation is a measure of the asymmetry of the distribution of CT density and represents the distribution pattern of CT attenuation in a histogram. A positive skewness value reflects a histogram with a tail longer on the right than on the left side. The higher positive skewness of CT attenuation observed in growing NSNs is consistent with a greater asymmetry of the CT attenuation distribution. The significant difference in skewness of CT attenuation between growing and nongrowing NSNs found in this study probably reflects the greater intralesional heterogeneity of growing NSNs compared to that of nongrowing NSNs. In other words, even if a pathological correlation was not performed in this study, we assume that the positive skewness observed in the growing NSNs is the result of the presence of some pixels with higher CT attenuation which could reflect the presence of more aggressive neoplastic foci.

The LMD is a quantitative feature that combines density and size measurements in a single value and it represents the two-dimensional variant of the mass [12, 13, 30]. As recently reported [30], LMD is a quantitative feature particularly appropriate to evaluate the characteristics and the behavior of SSNs on CT images, both at the baseline and during follow-up. In a previous retrospective study, Bak et al. [22] found that the 97.5th percentile of the mean CT attenuation and the slope of mean CT attenuation from the 2.5th to the 97.5th percentile, two other histogram-related CT features not tested in our study, were useful predictors of growth in a group of 54 NSNs. More recently, Sun et al. [23] showed that uniformity, another quantitative feature, is an effective predictor of growth in a group of 42 NSNs, with lower uniformity values in growing NSNs. Similar to skewness of CT attenuation, the 97.5th percentile of the mean CT attenuation, the slope of mean CT attenuation from the 2.5th to the 97.5th percentile, and uniformity reflect the intralesional heterogeneity of growing NSNs [22, 23]. Therefore, based on the findings of the present and the above previous studies, it can be stated with reasonable certainty that quantitative CT features reflecting intralesional heterogeneity are useful predictors of NSN growth.

We also found no significant correlation between NSN growth and certain quantitative features related to CT attenuation (i.e., mean, median, modal, and standard deviation of CT attenuation).

In a similar study performed on Asian (Chinese) patients, Shi et al. [24] reported that only the standard deviation of the CT attenuation value (greater than the cutoff value of 50 HU) and the maximum diameter (greater than the cutoff value of 10.2 mm) were independent predictors of growth in a group of 101 NSNs detected in 59 patients. The differences between ours and their findings could be attributable to several factors: the software-based method used (two-dimensional analysis with ImageJ versus volumetric analysis with 3D slicer); the quantitative CT features considered for the analysis; the method for defining the growing group (DT of LMD versus changes in maximum diameter and appearance of solid component); NSN selection criteria (only solitary NSNs were included in our study); patient ethnicity (Italian versus Chinese patients).

Prior studies have also reported that the mean CT attenuation value is an independent predictor of NSN growth [42, 43]; however, more recent studies, including our own, focused on software-based CT analysis of NSNs did not confirm the predictive value of this parameter [22–25].

Contrarily to previous studies [42, 43], we did not find any significant relationship between NSN growth and certain independent variables, including sex, smoking habits, history of cancer, and NSN location. Although the univariate analysis revealed a relationship between NSN growth, patient age, and the presence of pulmonary emphysema, this association was not confirmed in the multivariable analysis.

This study had some limitations. First, it was performed retrospectively and included a relatively small number of NSNs; however, our inclusion criteria were extremely strict and only solitary NSNs scanned with the same acquisition-reconstruction parameters and the same CT scanner were selected. Second, the software-based analysis was performed by a single radiologist; however, the reliability in measurement of the selected quantitative features was found to be excellent or good. Third, the quantitative CT analysis was performed only on two-dimensional axial images; however, the largest cross-sectional area should be the most representative image for assessing the internal characteristics and shape features of NSNs.

Despite these limitations, our study further highlights the potential role of software-based quantitative CT analysis, specifically the leading role of skewness of CT attenuation (along with the LMD), as a tool to improve risk stratification and devise personalized management plans for patients with pulmonary NSNs.

It is well known that most persistent NSNs exhibit an indolent course and may remain stable for several years [5, 20]; however, some NSNs grow quickly and require surgical removal [13]. Therefore, identifying quantitative CT features capable of early discrimination between growing and nongrowing NSNs is becoming a crucial aspect of radiological analysis to reduce the number of CT examinations and improve the timing of follow-up. From this clinical point of view, the application of software-based CT analysis to evaluate NSNs improve radiologist performance through deeper image analysis, highlighting CT features that cannot be assessed visually.

Obviously, the performance of software-based analysis and the promising predictive power of the skewness of CT attenuation observed in the present study need to be confirmed in future prospective analyses with larger sample sizes. However, we believe that computer-based quantitative CT analyses applied in NSNs (regardless of the software used) will have strong implications in the clinical setting, as they can affect the decision-making process and nodule management.

In conclusion, the results of the present study showed that the skewness of CT attenuation was the strongest predictor of growth in NSNs with an axial diameter of 6 − 30 mm detected in a Caucasian (Italian) population. According to our preliminary data, NSNs with a skewness value greater than 0.90, specifically those with a LMD > 19.16 mg/mm, should require closer follow-up due to their higher growth potential, and consequently, higher risk of becoming a clinically active cancer.

## References

[1] Kobayashi Y, Sakao Y, Deshpande GA, Fukui T, Mizuno T, Kuroda H, Sakakura N, Usami N, Yatabe Y, Mitsudomi T (2014). The association between baseline clinical-radiological characteristics and growth of pulmonary nodules with ground-glass opacity. Lung Cancer.

[2] Yip R, Yankelevitz DF, Hu M, Li K, Xu DM, Jirapatnakul A, Henschke CI (2016). Lung cancer deaths in the national lung screening trial attributed to nonsolid nodules. Radiology.

[3] Silva M, Prokop M, Jacobs C, Capretti G, Sverzellati N, Ciompi F, van Ginneken B, Schaefer-Prokop CM, Galeone C, Marchianò A, Pastorino U (2018). Long-term active surveillance of screening detected subsolid nodules is a safe strategy to reduce overtreatment. J Thorac Oncol.

[4] Rundo L, Ledda RE, di Noia C, Sala E, Mauri G, Milanese G, Sverzellati N, Apolone G, Gilardi MC, Messa MC, Castiglioni I, Pastorino U (2021). A low-dose CT-based radiomic model to improve characterization and screening recall intervals of indeterminate prevalent pulmonary nodules. Diagnostics.

[5] Borghesi A, Michelini S, Golemi S, Scrimieri A, Maroldi R (2020). What’s new on quantitative CT analysis as a tool to predict growth in persistent pulmonary subsolid nodules?. Lit Rev Diagn.

[6] MacMahon H, Naidich DP, Goo JM, Lee KS, Leung ANC, Mayo JR, Mehta AC, Ohno Y, Powell CA, Prokop M, Rubin GD, Schaefer-Prokop CM, Travis WD, Van Schil PE, Bankier AA (2017). Guidelines for management of incidental pulmonary nodules detected on CT images: from the fleischner society 2017. Radiology.

[7] Bueno J, Landeras L, Chung JH (2018). Updated fleischner society guidelines for managing incidental pulmonary nodules: common questions and challenging scenarios. Radiographics.

[8] Choi WS, Park CM, Song YS, Lee SM, Wi JY, Goo JM (2015). Transient subsolid nodules in patients with extrapulmonary malignancies: their frequency and differential features. Acta Radiol.

[9] Chung K, Ciompi F, Scholten ET, Goo JM, Prokop M, Jacobs C, van Ginneken B, Schaefer-Prokop CM (2018). Visual discrimination of screen-detected persistent from transient subsolid nodules: an observer study. PLoS ONE.

[10] Huang C, Lv W, Zhou C, Mao L, Xu Q, Li X, Qi L, Xia F, Li X, Zhang Q, Zhang L, Lu G (2020). Discrimination between transient and persistent subsolid pulmonary nodules on baseline CT using deep transfer learning. Eur Radiol.

[11] Ricciardi S, Booton R, Petersen RH, Infante M, Scarci M, Veronesi G, Cardillo G (2021). Managing of screening-detected sub-solid nodules-a European perspective. Transl Lung Cancer Res.

[12] Song YS, Park CM, Park SJ, Lee SM, Jeon YK, Goo JM (2014). Volume and mass doubling times of persistent pulmonary subsolid nodules detected in patients without known malignancy. Radiology.

[13] Borghesi A, Farina D, Michelini S, Ferrari M, Benetti D, Fisogni S, Tironi A, Maroldi R (2016). Pulmonary adenocarcinomas presenting as ground-glass opacities on multidetector CT: Three-dimensional computer-assisted analysis of growth pattern and doubling time. Diagn Interv Radiol.

[14] Lee JH, Park CM, Lee SM, Kim H, McAdams HP, Goo JM (2016). Persistent pulmonary subsolid nodules with solid portions of 5 mm or smaller: their natural course and predictors of interval growth. Eur Radiol.

[15] Albano D, Borghesi A, Bosio G, Bertoli M, Maroldi R, Giubbini R, Bertagna F (2017). Pulmonary mucosa-associated lymphoid tissue lymphoma: (18) F-FDG PET/CT and CT findings in 28 patients. Br J Radiol.

[16] Cozzi D, Dini C, Mungai F, Puccini B, Rigacci L, Miele V (2019). Primary pulmonary lymphoma: imaging findings in 30 cases. Radiol Med.

[17] Borghesi A, Tironi A, Michelini S, Scrimieri A, Benetti D, Maroldi R (2019). Two synchronous lung metastases from malignant melanoma: the same patient but different morphological patterns. Eur J Radiol Open.

[18] Borghesi A, Bercich L, Michelini S, Bertagna F, Scrimieri A, Maroldi R (2019). Pulmonary metastases from malignant epithelioid schwannoma of the arm presenting as fast-growing subsolid nodules: report of an unusual case. Eur J Radiol Open.

[19] American College of Radiology (2019) Lung CT screening reporting and data system (Lung-RADS) Version 1.1 Assessment Categories (2019 release). Available online: https://www.acr.org. Accessed 2 August 2022

[20] Kobayashi Y, Mitsudomi T (2013). Management of ground-glass opacities: should all pulmonary lesions with ground-glass opacity be surgically resected?. Transl Lung Cancer Res.

[21] Gao C, Li J, Wu L, Kong D, Xu M, Zhou C (2020). The natural growth of subsolid nodules predicted by quantitative initial CT features: a systematic review. Front Oncol.

[22] Bak SH, Lee HY, Kim JH, Um SW, Kwon OJ, Han J, Kim HK, Kim J, Lee KS (2016). Quantitative CT scanning analysis of pure ground-glass opacity nodules predicts further CT scanning change. Chest.

[23] Sun Q, Huang Y, Wang J, Zhao S, Zhang L, Tang W, Wu N (2019). Applying CT texture analysis to determine the prognostic value of subsolid nodules detected during low-dose CT screening. Clin Radiol.

[24] Shi Z, Deng J, She Y, Zhang L, Ren Y, Sun W, Su H, Dai C, Jiang G, Sun X, Xie D, Chen C (2019). Quantitative features can predict further growth of persistent pure ground-glass nodule. Quant Imaging Med Surg.

[25] Qi LL, Wu BT, Tang W, Zhou LN, Huang Y, Zhao SJ, Liu L, Li M, Zhang L, Feng SC, Hou DH, Zhou Z, Li XL, Wang YZ, Wu N, Wang JW (2019). Long-term follow-up of persistent pulmonary pure ground-glass nodules with deep learning-assisted nodule segmentation. Eur Radiol.

[26] Borghesi A, Michelini S, Bertagna F, Scrimieri A, Pezzotti S, Maroldi R (2018). Hilly or mountainous surface: a new CT feature to predict the behavior of pure ground glass nodules?. Eur J Radiol Open.

[27] Gao C, Yan J, Luo Y, Wu L, Pang P, Xiang P, Xu M (2020). The growth trend predictions in pulmonary ground glass nodules based on radiomic CT features. Front Oncol.

[28] Lui NS, Benson J, He H, Imielski BR, Kunder CA, Liou DZ, Backhus LM, Berry MF, Shrager JB (2020). Sub-solid lung adenocarcinoma in Asian versus Caucasian patients: different biology but similar outcomes. J Thorac Dis.

[29] Rasband WS (1997–2018) ImageJ, U.S. National Institutes of Health, Bethesda, Maryland, USA. Available online: https://imagej.nih.gov/ij/. Accessed 6 July 2020

[30] Borghesi A, Scrimieri A, Michelini S, Calandra G, Golemi S, Tironi A, Maroldi R (2019). Quantitative CT analysis for predicting the behavior of part-solid nodules with solid components less than 6 mm: size. Density Shape Descr Appl Sci.

[31] Wu YJ, Wu FZ, Yang SC, Tang EK, Liang CH (2022). Radiomics in early lung cancer diagnosis: from diagnosis to clinical decision support and education. Diagnostics.

[32] Nemec U, Heidinger BH, Anderson KR, Westmore MS, VanderLaan PA, Bankier AA (2018). Software-based risk stratification of pulmonary adenocarcinomas manifesting as pure ground glass nodules on computed tomography. Eur Radiol.

[33] Borghesi A, Michelini S, Nocivelli G, Silva M, Scrimieri A, Pezzotti S, Maroldi R, Farina D (2019). Solid indeterminate pulmonary nodules less than or equal to 250 mm^3^: application of the updated fleischner society guidelines in clinical practice. Radiol Res Pract.

[34] Borghesi A, Michelini S, Scrimieri A, Golemi S, Maroldi R (2019). Solid indeterminate pulmonary nodules of less than 300 mm^3^: application of different volume doubling time cut-offs in clinical practice. Diagnostics.

[35] Qiu L, Zhang X, Mao H, Fang X, Ding W, Zhao L, Chen H (2022). Comparison of comprehensive morphological and radiomics features of subsolid pulmonary nodules to distinguish minimally invasive adenocarcinomas and invasive adenocarcinomas in CT Scan. Front Oncol.

[36] Qi L, Lu W, Yang L, Tang W, Zhao S, Huang Y, Wu N, Wang J (2019). Qualitative and quantitative imaging features of pulmonary subsolid nodules: differentiating invasive adenocarcinoma from minimally invasive adenocarcinoma and preinvasive lesions. J Thorac Dis.

[37] Feng B, Chen X, Chen Y, Li Z, Hao Y, Zhang C, Li R, Liao Y, Zhang X, Huang Y, Long W (2019). Differentiating minimally invasive and invasive adenocarcinomas in patients with solitary sub-solid pulmonary nodules with a radiomics nomogram. Clin Radiol.

[38] Cohen JG, Reymond E, Medici M, Lederlin M, Lantuejoul S, Laurent F, Toffart AC, Moreau-Gaudry A, Jankowski A, Ferretti GR (2018). CT-texture analysis of subsolid nodules for differentiating invasive from in-situ and minimally invasive lung adenocarcinoma subtypes. Diagn Interv Imaging.

[39] Fan L, Fang M, Li Z, Tu W, Wang S, Chen W, Tian J, Dong D, Liu S (2019). Radiomics signature: a biomarker for the preoperative discrimination of lung invasive adenocarcinoma manifesting as a ground-glass nodule. Eur Radiol.

[40] Liang X, Liu M, Li M, Zhang L (2022). Clinical and CT features of subsolid pulmonary nodules with interval growth: a systematic review and meta-analysis. Front Oncol.

[41] Sun Y, Ma Z, Zhao W, Jin L, Gao P, Wang K, Huang X, Duan S, Li M (2023). Computed tomography radiomics in growth prediction of pulmonary ground-glass nodules. Eur J Radiol.

[42] Eguchi T, Kondo R, Kawakami S, Matsushita M, Yoshizawa A, Hara D, Matsuoka S, Takeda T, Miura K, Agatsuma H, Sakaizawa T, Tominaga Y, Saito G, Toishi M, Hamanaka K, Hashizume M, Shiina T, Amano J, Koizumi T, Computed YK (2014). Computed tomography attenuation predicts the growth of pure ground-glass nodules. Lung Cancer.

[43] Tamura M, Shimizu Y, Yamamoto T, Yoshikawa J, Hashizume Y (2014). Predictive value of one-dimensional mean computed tomography value of ground-glass opacity on high-resolution images for the possibility of future change. J Thorac Oncol.

